# Copolymeric Micelles Overcome the Oral Delivery Challenges of Amphotericin B

**DOI:** 10.3390/ph13060121

**Published:** 2020-06-11

**Authors:** Pataranapa Nimtrakul, Desmond B. Williams, Waree Tiyaboonchai, Clive A. Prestidge

**Affiliations:** 1 Faculty of Pharmaceutical Sciences, Naresuan University, Phitsanulok 65000, Thailand; goofee_e@hotmail.com; 2 Clinical and Health Sciences, University of South Australia, Adelaide, SA 5000, Australia; Des.Williams@unisa.edu.au; 3 The Center of Excellence for Innovation in Chemistry (PERCH-CIC), Department of Chemistry, Faculty of Science, Mahidol University, Bangkok 10400, Thailand; 4 ARC Centre of Excellence in Convergent Bio-Nano Science & Technology, University of South Australia, Adelaide, SA 5000, Australia

**Keywords:** amphotericin B, Soluplus^®^, polymeric micelles, oral absorption, caco-2 cells, cellular uptake

## Abstract

Classified as a Biopharmaceutical Classification System (BCS) class IV drug, amphotericin B (AmB) has low aqueous solubility and low permeability leading to low oral bioavailability. To improve these limitations, this study investigated the potential of AmB-loaded polymeric micelles (AmB-PM) to increase intestinal absorption. AmB-PM were prepared with polyvinyl caprolactam–polyvinyl acetate–polyethylene glycol copolymer (Soluplus^®^) as a polymeric carrier and used a modified solvent diffusion and microfluidics (NanoAssemblr^®^) method. AmB-PM have a mean particle size of ~80 nm and are mono-disperse with a polydispersity index <0.2. The entrapment efficiency of AmB was up to 95% and achieved with a high drug loading up to ~20% (*w/w*) with a total amount of incorporated drug of 1.08 ± 0.01 mg/mL. Importantly, compared to free drug, AmB-PM protected AmB from degradation in an acidic (simulated gastric) environment. Viability studies in Caco-2 cells confirmed the safety/low toxicity of AmB-PM. In vitro cellular absorption studies confirmed that AmB-PM increased AmB uptake in Caco-2 cells 6-fold more than free AmB (i.e., 25% compared with 4% within 30 min). Furthermore, the permeability of AmB across Caco-2 monolayers was significantly faster (2-fold) and more pronounced for AmB-PM in comparison to free drug (3.5-fold increase). Thus, the developed AmB-PM show promise as a novel oral delivery system for AmB and justifies further investigation.

## 1. Introduction

Amphotericin B (AmB) is the first effective antifungal agent and is considered one of the gold standards for the treatment of systemic fungal infections due to its broad-spectrum activity and relatively low resistance against most fungal infections. AmB is highly effective against several fungal organisms, including *Candida species, Aspergillus fumigatus, Histoplasma capsulatum*, or *Cryptococcus neoformans* [1,2]. However, it is classified as a Biopharmaceutical Classification System class IV drug and regarded as a poor candidate for oral formulation development due to its low aqueous solubility, low permeability, as well as its instability in acidic environments (pH < 2) [3]. As a consequence, no oral AmB products are available in the market, only products for intravenous administrations. Although parenteral administration would provide faster drug onset than oral administration, it is inconvenient, requires aseptic techniques, is associated with pain or discomfort, and is more expensive [4,5]. Compared to the parenteral route, the oral route is the most widely used and acceptable because it can be self-administered with lower cost, is convenient, and painless, which offers greater patient acceptance and compliance.

Therefore, oral drug delivery systems for AmB have been widely explored recently. Among these systems, nanocarriers have been extensively investigated, including solid-lipid nanoparticles (SLNs) or nanostructured lipid carriers (NLCs) [6,7], O/W Microemulsion [8], polymeric nanoparticles [9,10,11], chitosan-coated NLCs [12], cubosomes [13,14], carbon nanotubes [15], and self-emulsifying drug delivery systems (SEDDS) [16,17,18]. They could improve AmB oral bioavailability by increasing AmB water solubility and protecting the drug from acidic environments. Unfortunately, most of these nanocarriers have some limitations involving low drug loading capacity and in vivo toxicity.

To overcome these limitations, we proposed polymeric micelles (PM) as a nanocarrier for oral AmB. PM are obtained by self-assembly of amphiphilic polymers in water at concentrations higher than the critical micellar concentration (CMC). They possess a hydrophobic core–hydrophilic shell architecture that facilitates the loading of hydrophobic drugs into the core, while the outer hydrophilic shell protects the drug from degradation in gastrointestinal (GI) fluid [19,20,21]. Moreover, drug-loaded PM with a size of <100 nm are reported to improve drug transport across intestinal epithelium via endocytosis [22,23,24,25]. Among amphiphilic polymers, PM prepared from Soluplus^®^ have been investigated to enhance the oral bioavailability of poorly soluble drugs with high drug loading. Soluplus^®^ is a non-ionic, amphiphilic, grafted copolymer composed of a polyvinyl, caprolactam–polyvinyl, and acetate–polyethylene glycol at a ratio of 57/30/13. Soluplus^®^ has an excellent matrix-forming ability with a critical micelle concentration of ~0.0076 mg/mL and is non-ionic, which offers pH-independent solubility [26,27].

This present study aimed to develop AmB-loaded polymeric micelles (AmB-PM) for oral administration. The physicochemical properties, including size, charge, drug loading capacity, and entrapment efficiency, were investigated. The chemical stability of AmB-PM was also examined in bio relevant media by simulating the fasting stomach and intestine. Then, a Caco-2 in vitro model was applied to predict the oral bioavailability of AmB-PM. The cellular uptake and transport of AmB-PM were compared to the free drug. An in vitro cytotoxicity of prepared AmB-PM was also investigated.

## 2. Results

### 2.1. Physicochemical Properties of AmB–Soluplus^®^ Micelles

**Figure 1:**
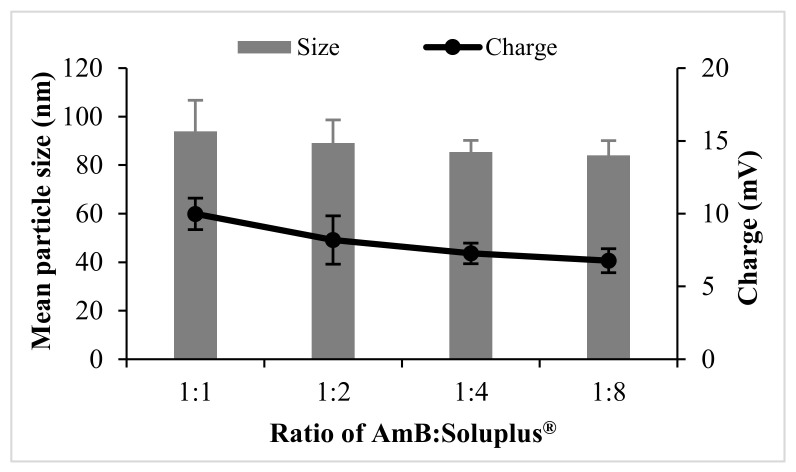
Particle size and charge of amphotericin B-loaded polymeric micelles (AmB-PM) prepared with different weight ratios of drug and polymers (*n* = 3).

**Figure 2:**
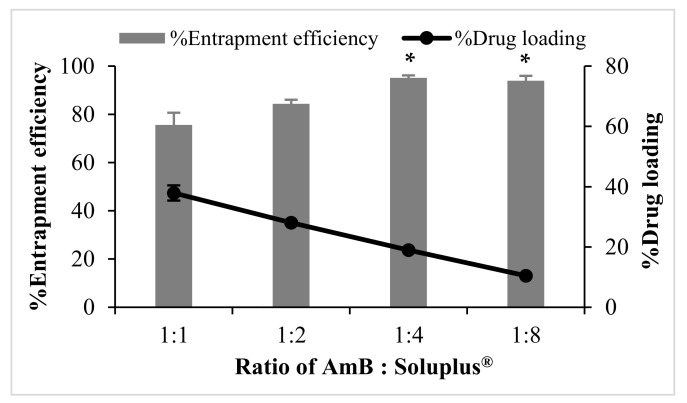
Entrapment efficiency (%EE) and drug loading (%DL) of AmB-PM prepared with different weight ratios of drug and polymers (*n* = 3). Data are presented as mean ± SD, (*n* = 3). * indicate AmB-PM (1:4) and (1:8) significantly different to AmB-PM (1:1) and (1:2) (*p* < 0.05).

The mean particle size and charge of AmB-PM were found to be independent of the ratio of drug and Soluplus^®^. All formulations possessed a mean size of ~80–90 nm, with a narrow size distribution, polydispersity index (PDI) <0.2, and demonstrated a positive charge of ~8 mV, see Figure 1. Blank Soluplus^®^ micelles showed a slightly smaller particle size of ~60 to 70 nm (data not shown). Drug loading (%DL) and entrapment efficiency (%EE) were related to the polymer concentrations, see Figure 2. When increasing Soluplus^®^ content, %DL was decreased, while %EE was increased. %EE of AmB and Soluplus^®^ at 1:1 and 1:2 ratio showed no significant differences (~76% and ~84%, respectively), while %EE of AmB and Soluplus^®^ at 1:4 and 1:8 ratio were significantly increased to ~94%.

### 2.2. Chemical Stability of AmB-Soluplus^®^ Micelles in GI Fluids

**Figure 3:**
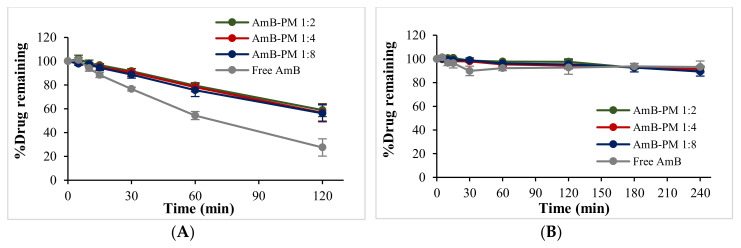
%Drug remaining of AmB-PM and free drug in (**A**) fasted state simulated gastric fluid (FaSSGF) pH 1.6 and (**B**) fasted state simulated intestinal fluid (FaSSIF) pH 6.5 (*n* = 3).

The degradation of AmB was investigated in fasted state simulated gastric fluid pH 1.6 (FaSSGF) and fasted state simulated intestinal fluid pH 6.5 (FaSSIF) and shown in Figure 3A,B, respectively. In FaSSGF, free AmB exhibited rapid degradation, ~50% and ~30% of the drug remaining after 1 h and 2 h of incubation, respectively, while AmB-PM retained more than 60% of AmB after 2 h incubation. The stability of AmB showed no significant differences with increasing Soluplus^®^ proportions. As expected, both free drug and AmB-PM showed no AmB degradation in FaSSIF. The degradation half-life of the free drug and AmB-PM was calculated to be ~75 min and 140 min, respectively.

### 2.3. Cytotoxicity of AmB-Soluplus^®^ Micelles in Caco-2 Cells

**Figure 4:**
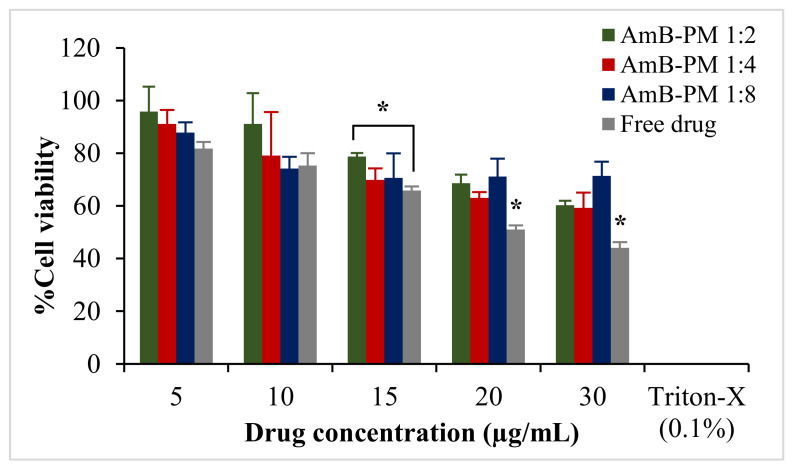
Viability of Caco-2 cells after incubation with AmB-PM and free drug for 4 h. Data are presented as mean ± SD, (*n* = 3). * indicate statistically significant differences between formulations (*p* < 0.05).

AmB-PM exhibited Caco-2 cell viability of >70% at a drug concentration ranging from 5 to 20 μg/mL, Figure 4. However, free drug exhibited a higher toxic effect. At AmB concentrations of 20 μg/mL and 30 μg/mL the cell viabilities were <50%. According to the International Organization for Standardization (ISO 10993-5) [28], 70% is commonly accepted as the threshold for cell viability. Therefore, 15 μg/mL was considered the highest drug concentration for the subsequent cell uptake and transport studies. The cytotoxicity profile of the blank PM at all the concentrations that correspond to AmB-PM showed no potential cytotoxicity to the cells; the cell viabilities were >90% (data not shown).

### 2.4. Cellular Uptake of AmB-Soluplus^®^ Micelles

**Figure 5:**
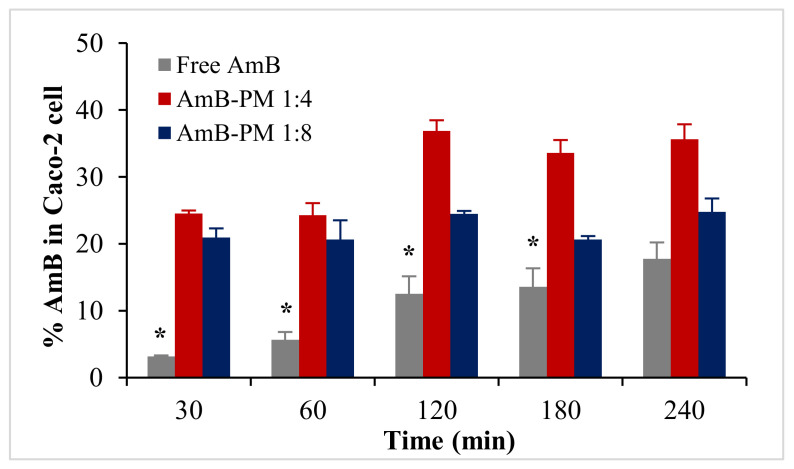
Uptake of AmB (%) in Caco-2 cells treated with AmB-PM and free drug. Data are presented as mean ± SD, (*n* = 3). * indicate statistically significant differences between formulations (*p* < 0.05).

Based on the %EE results, two formulations, AmB-PM (1:4) and AmB-PM (1:8), were selected for cellular uptake and transport studies (the 1:1 and 1:2 formulations were less stable and had high fractions of the non-encapsulated drug). Uptake of AmB-PM in Caco-2 cells is represented in Figure 5. Both AmB-PM (1:4) and (1:8) showed significantly greater and faster cellular uptake (*p* < 0.05) than the free drug. Uptake of free drug increased almost linearly with time. In contrast, AmB-PM (1:4) showed faster cellular uptake of ~25% within 30 min and increased to ~37% within 2 h. Whereas AmB-PM (1:8) showed ~20% cellular uptake within 30 min then, the drug remained constant until 4 h.

### 2.5. Cellular Transport of AmB-Soluplus^®^ Micelles Across Caco-2 Cell Monolayer

**Figure 6:**
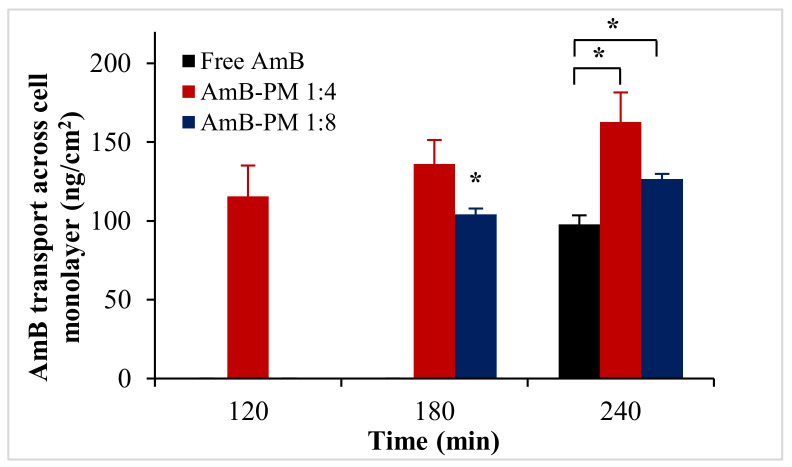
AmB transport across Caco-2 cell monolayer after incubation with AmB-PM and free drug. Data are presented as mean ± SD, (*n* = 3). * indicate statistically significant differences between formulations (*p* < 0.05).

**Figure 7:**
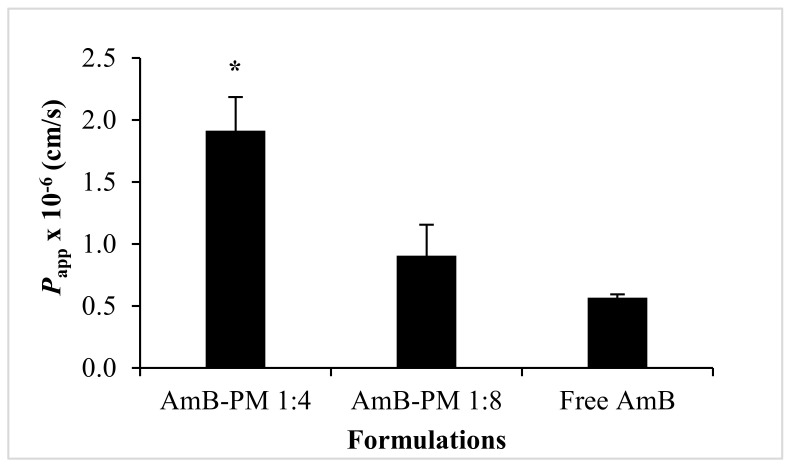
The corresponding *P*_app_ values of AmB-PM across the Caco-2 cell monolayer. Data are presented as mean ± SD, (*n* = 3). * indicate statistically significant differences between groups (*p* < 0.05).

Transportation of AmB-PM and free AmB across Caco-2 cell monolayers is shown in Figure 6. AmB-PM exhibited faster drug transport across Caco-2 cell monolayers than free drug. For AmB-PM (1:4) and (1:8), AmB was initially detected in the basolateral chamber after 2 h and 3 h of incubation, respectively. However, for free drug, AmB was detected after 4 h of incubation. In addition, after 4 h of incubation, the concentrations of AmB transport from AmB-PM (1:4) (~162 ng/cm^2^) and AmB-PM (1:8) (~126 ng/cm^2^) were significantly higher than free drug (~97 ng/cm^2^). However, the limit of quantitation (LOQ) and limit of detection (LOD) of AmB were found to be 100 ng/mL and 50 ng/mL, respectively. During the experiments, only 0.2 mL of basolateral medium was withdrawn; therefore, the amount of drug in the basolateral chamber might be below the limit of detection. The corresponding *P*_app_ values were calculated based on the ability of drug transportation across the cell monolayer and are shown in Figure 7. The *P*_app_ value of AmB-PM (1:4) (~1.91 × 10^−6^ cm/s) was 3.5-fold and 2-fold higher than those of free drug (~0.57 × 10^−6^ cm/s) and AmB-PM (1:8) (~0.91 × 10^−6^ cm/s), respectively. These values are in agreement with Yee et al., who reported a *P*_app_ value between 1 × 10^−6^ cm/s and 10 × 10^−6^ cm/s, which corresponded to moderate absorption (20–70%) [29]. According to the *P*_app_ values, AmB-PM were classified as having moderate permeability.

**Figure 8:**
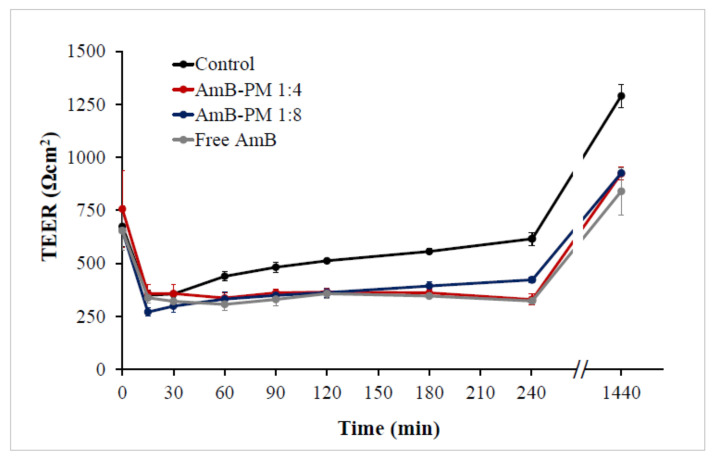
Transepithelial electrical resistance (TEER) values of Caco-2 cells after incubation with AmB-PM and free drug, immediately after the experiment or following 24 h recovery in standard growth media. (*n* = 3).

To ensure cell monolayer integrity, the transepithelial electrical resistance (TEER) values were monitored before, during, and after the transportation studies. The initial (control) monolayer TEER values of 650 to 750 Ω.cm^2^ decreased to ~300 Ω.cm^2^ within 15 min of AmB addition, see Figure 8. It has been reported that the Caco-2 cell monolayers that generate a TEER of 150 to 400 Ω.cm^2^ are sufficient to restrict the diffusion of substances across the barrier [30]. Thus, the cell monolayer remained intact during transport studies. Moreover, full recovery of the TEER value was observed in all samples after the solutions were replaced with standard growth media and incubated for another 24 h. The reduction in TEER values in AmB-PM is considered a result of a transient disruption of the Caco-2 cell monolayer integrity from the amphiphilic nature of AmB and Soluplus^®^ micelles that are able to increase the fluidity of the membrane or loosen tight junctions [31].

### 2.6. Cellular Uptake of Nile Red-Soluplus^®^ Micelles

**Figure 9:**
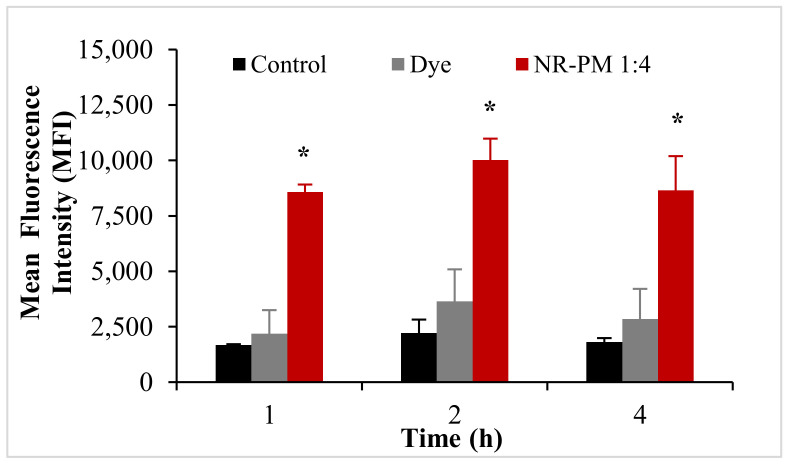
Mean fluorescence intensity of Nile red (NR)-PM in Caco-2 cells. Data are presented as mean ± SD, (*n* = 3). * indicate statistically significant differences between groups (*p* < 0.05).

Based on the results from the cellular uptake and transport studies, AmB-PM (1:4) showed the highest amount of drug uptake and transport in Caco-2 cells. Thus, Nile red (NR)-PM (1:4) was selected to quantify and visualize the cellular uptake of Soluplus^®^ micelles. The cellular uptake of NR-PM per cell is represented by the mean fluorescence intensity (MFI) of positive cells, see Figure 9. No significant differences in MFI values were observed between the control groups. As expected, the MFI of cells treated with NR-PM were significantly (3-fold) higher than those of control groups.

**Figure 10:**
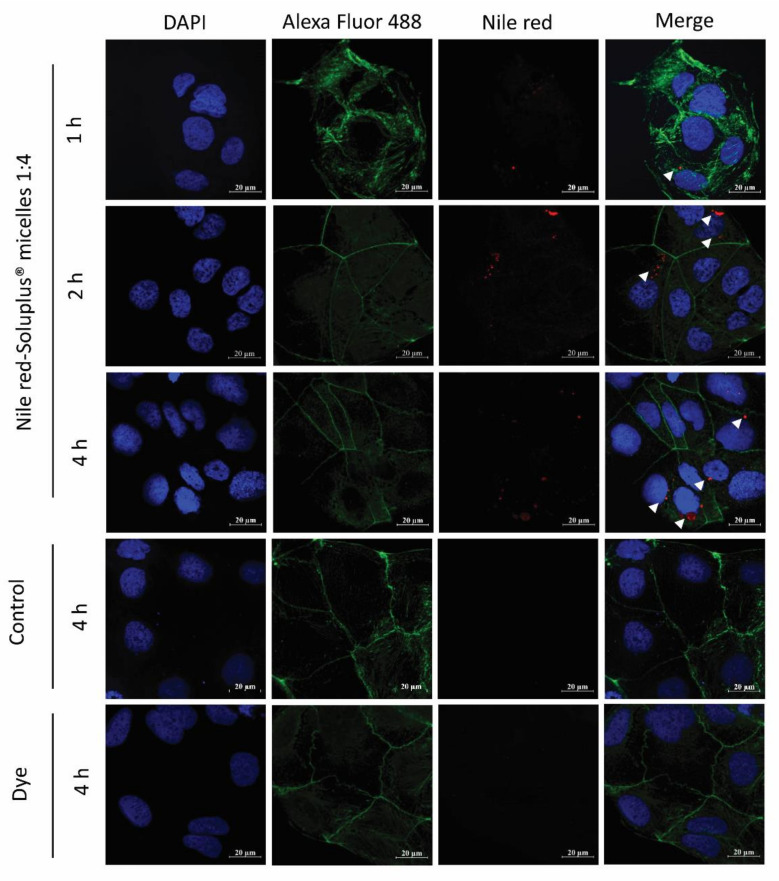
Confocal laser scanning microscopy images of Caco-2 cells with NR-PM (1:4); arrowheads indicate the presence of the micelles within the cells. Red: NR-PM, blue: nucleus, green: cell membrane. Magnification 40×; scale bar = 20 μm.

Cellular uptake of NR-PM was also confirmed by confocal laser scanning microscopy (CLSM). The cells treated with NR-PM (1:4) exhibited an intracellular fluorescence signal of Nile red, Figure 10. The fluorescent signals of Nile red in cells were observed to become gradually stronger at 1, 2, and 4 h, while no fluorescence signal of Nile red was observed in cells treated with culture media (negative control) and Nile red solution (positive control) at all incubation times.

## 3. Discussion

AmB-PM were successfully prepared by a modified solvent diffusion method using the NanoAssemblr^®^ Benchtop instrument with a high drug loading of ~20%. AmB-PM were spontaneously formed when the organic phase was mixed with the aqueous phase. The Soluplus^®^ amphiphilic block copolymers were assembled into spherical core–shell micelles, which consisted of a hydrophobic core for AmB loading and a hydrophilic shell. The process parameters, such as different weight ratios of AmB and Soluplus^®^, had no significant effect on the particle size and charge. The developed AmB-PM possessed a similar mean particle size of ~80 nm with PDI less than 0.2, indicating mono-disperse and homogenous sizes. Particle size plays a key role in GI absorption. It has been reported that PM with a size of <100 nm could promote intestinal absorption through endocytosis [32,33,34]. In general, Soluplus^®^ is a non-ionic polymer, and, thus, Soluplus^®^ micelles possess no surface charge. AmB-PM showed a positive charge of ~+8 mV. This was due to the final pH of the systems (pH ~4.5) being less than the pKa of AmB (~5.7) [35]. Thus, AmB showed a positive charge that comes from the AmB amine group. AmB-PM showed a positive charge of < 30 mV, suggesting no strong electrostatically stabilizing effect that may lead to particle aggregation [36,37,38]. However, AmB-PM were physically stable, and no precipitation was observed, which is considered a result of the steric stabilizing effect through micelle hydrophilic shell. The temporal stability of AmB-PM (1:4) and AmB-PM (1:8) were preliminary evaluated at 4 °C in the absence of daylight. After 1-month storage, AmB-PM (1:4) and AmB-PM (1:8) showed the percent drug remaining of more than 90%. The mean particle size and charge of the AmB-PM of tested formulations showed no significant difference as compared to those of initial preparations (Table S1 in Supplementary Material). Thus, it is reasonably demonstrated that AmB-PM has good physical and chemical stability for, at least, 1-month storage at 4 °C. In addition, AmB-PM (1:4) provided high drug loading up to 20% with the total amount of incorporated drug of 1.08 ± 0.01 mg/mL.

AmB is unstable in acid conditions, which is one of the major challenges for its oral delivery. Thus, the ability of AmB-PM to protect AmB degradation in GI fluids was investigated and compared to free drug using the biorelevant media, FaSSGF and FaSSIF. As expected, after 2 h of incubation in FaSSGF, AmB-PM exhibited more than 2-fold of AmB remaining (~60%) compared to free drug (~30%). The protective effect of Soluplus^®^ micelles could possibly be explained by AmB being localized in a micelle hydrophobic core and enclosed by a hydrophilic shell, which helped limit the drug’s direct contact with the acidic environment.

To confirm AmB-PM had potential in enhancing oral bioavailability of AmB, the in vitro absorption of AmB-PM was investigated in Caco-2 cells. These cells are the most widely used for predicting drug–intestinal permeability because the Caco-2 cell monolayer resembles the human intestinal barrier in morphology, polarity, and expression patterns of transporters and enzymes [39,40]. AmB is not a substrate for P-glycoprotein (P-gp) [41]. Thus, only drug transport from the apical to the basolateral chamber was investigated. AmB-PM and free drug were added through the apical chamber, and samples were collected accordingly from the basolateral chamber. As expected, Soluplus^®^ micelles proved to be effective for enhancing AmB permeability more than free drug. The result illustrated that AmB-PM demonstrated significantly faster and greater AmB transport into the cell and across cell monolayers than the free drug.

AmB, an amphiphilic molecule, is poorly water soluble with a high molecular weight of ~924 Da and a log *P* value of 0.8. It could transport into the cell via a transcellular pathway by diffusion and where the transport rate is dependent on the drug concentration [42]. As a result, slow drug diffusion into cells was observed, with only 4% of AmB found in Caco-2 cells after 30 min of incubation. In addition, AmB could have sufficiently strong interactions with cell membrane sterols [43] in the apical side, and, thus, take a longer time, 4 h, to reach the basolateral chamber.

On the contrary, the increase in transport of AmB-PM could be explained by two factors. First, the low water solubility of AmB is a major barrier to prevent passive absorption. Therefore, with the incorporation of AmB into Soluplus^®^ micelles, AmB is well dispersed in water with high drug concentration gradients readily available for diffusion into Caco-2 cells. Second, in accordance with the hypothesis that AmB-PM internalized into cells via endocytosis, due to their particle size of ~80 nm, it was advantageous for enhancing drug transport via this pathway. It has been reported that particles with sizes below 100 nm showed optimum cellular uptake into epithelial cells, which is desirable for transport into the cytoplasm, and exocytosed their contents into the blood circulation [24,25]. Therefore, AmB-PM showed higher AmB in Caco-2 cells, 25% within 30 min, and took a shorter time, 2 h, to transport AmB to the basolateral chamber. Comparing AmB-PM (1:4) and AmB-PM (1:8), AmB-PM (1:4) showed two times greater cellular uptake and faster drug transport across Caco-2 cell monolayers. These results are influenced by the drug loading capacity. AmB-PM (1:4) possessed two times higher drug loading, 20%, as compared to AmB-PM (1:8), 11%, leading to endocytosis of higher drug solubilization in micelles, followed by exocytosis into the basolateral chamber. Furthermore, the cellular uptake of NR-PM demonstrated the competency of internalization of NR-PM, implying that the uptake and transport of developed micelles occurred through a transcellular pathway. In addition, Hu Mei et al. reported that the absorption of dabigatran etexilate, when formulated in Soluplus^®^ micelles, were found to be internalized via an endocytosis pathway [33]. Such findings lend support to the hypothesis that AmB- Soluplus^®^ micelles could be internalized into cells via this pathway. Considering all data, the AmB-PM have the potential to improve the in vitro efficacy of AmB for oral delivery by protecting the drug from degradation and enhancing drug absorption in the GI tract.

## 4. Materials and Methods

### 4.1. Materials and Regents

The chemicals and sources for purchase were Standard AmB. Eighty percent Pure HPLC grade (Sigma-Aldrich, St. Louis, MO, USA), AmB, USP grade was (Biobasic Inc, Markham, ON, Canada), mannitol, sodium dihydrogen phosphate dihydrate, sodium acetate (Ajax Finechem Pty Ltd., New South Wales, Australia), simulated intestinal fluid (SIF) powder (Biorelevant, London, UK), dimethyl sulfoxide (DMSO) (BD Biosciences, New South Wales, Australia), HPLC grade organic solvents, including acetonitrile and methanol (Merck, Darmstadt, Germany), phosphate buffer solution (PBS) tablets, and D-α-tocopheryl polyethylene glycol succinate (VitE-TPGS) (Sigma-Aldrich, NSW, Australia), Alexa Fluor 488 conjugate and 3-(4,5-Dimethylthiazol-2-yl)-2,5-Diphenyltetrazolium Bromide (MTT) (Invitrogen, Carlsbad, CA, USA), glacial acetic acid, hydrochloric acid and sodium chloride (Chem-supply (Gillman, SA, Australia), CaCo-2 (c2bbe clone, ATCC^®^ CRL-2102™) cells (American Type Culture Collection (ATCC), Manassas, VA, USA), Hanks balanced salt solution (HBSS), phosphate buffered solution (PBS), Dulbecco’s modified Eagle medium (DMEM), fetal bovine serum (FBS), and Penicillin–Streptomycin (PS) (Thermo Fisher, Victoria, Australia). Soluplus^®^ was kindly supplied by BASF (Ludwigshafen, Germany). High purity Milli Q water (Merck Millipore, NSW, Australia) were used throughout the study.

### 4.2. Preparation of AmB-Soluplus^®^ Micelles

AmB-PM were prepared using the NanoAssemblr^®^ Benchtop instrument (Precision Nanosystems, Vancouver, BC, Canada). Briefly, the Soluplus^®^ and AmB were dissolved in an organic phase, a mixture of methanol and 0.1 M HCl at a volume ratio of 60:1. Then, the organic phase was injected into the first inlet and an aqueous phase (Milli-Q water) into the second inlet of the NanoAssemblr^®^. The total flow rate was held at 10 mL/min with a flow rate ratio of organic to aqueous phase of 1:1, and control total volume of 8 mL. The resulting polymeric micelles formed by the mixing of the two adjacent streams were collected from the outlet, and the methanol removed by stirring at 500 rpm overnight at room temperature. The resulting polymeric micelles were adjusted into a final volume of 5 mL with Milli-Q water and filtrated using PTFE 0.45 μm filter membrane to remove the residual aggregates. In this study, AmB-PM were prepared by varying the ratio of drug and polymer at 1:1, 1:2, 1:4, and 1:8, while the amount of AmB was kept constant at 8 mg.

### 4.3. Particle Size and Polydispersity Index Analysis

Mean particle size, polydispersity index (PDI) and charge of AmB-PM were measured by a Zetasizer NanoZS90 (Malvern Instruments, Malvern, UK). The percentages of entrapment efficiency (%EE) and drug loading (%DL) were determined by HPLC analysis (Shimadzu, Japan with a HALO C18 column, 5 μm, 4.6 × 150 mm, USA). To dissolve the micelles, AmB-PM were dissolved in methanol at ratio 1:10, diluted with a mobile phase, then injected into the HPLC system. Each sample was determined in triplicate. Ten-micromolar acetate buffer pH 5 and acetonitrile at a volume ratio of 65:35 was used as a mobile phase. The effluences were analyzed using a UV detector at 408 nm with a flow rate of 0.8 mL/min. The concentrations of AmB were quantified with a linear calibration curve using the peak area over the range of 0.04 to 8 μg/mL. The %EE and %DL were calculated according to Equations (1) and (2), respectively.

(1) $$\%\mathrm{EE}=\frac{\mathrm{Amount}\;\mathrm{of}\;\mathrm{drug}\;\mathrm{detected}}{\mathrm{Initial}\;\mathrm{amount}\;\mathrm{of}\;\mathrm{drug}}\;\times \;100$$

(2) $$\%\mathrm{DL}=\frac{\mathrm{Amount}\;\mathrm{of}\;\mathrm{drug}\;\mathrm{detected}}{\mathrm{Amount}\;\mathrm{of}\;\mathrm{polymer}\;\mathrm{and}\;\mathrm{drug}\;\mathrm{content}}\;\times \;100$$

### 4.4. Chemical Stability of AmB-Soluplus^®^ Micelles in Gastrointestinal Fluids

The chemical stability of AmB-PM was determined in FaSSGF and FaSSIF. AmB-PM (3 mg drug) were dispersed in 300 mL of simulated fluid. The mixture was maintained at 37 °C with a paddle speed of 50 rpm using a Vankel USP II paddle apparatus (Agilent Technologies, Santa Clara, CA, USA). Stirring was continued for 2 h in FaSSGF and 4 h in FaSSIF. Free drug dissolved in DMSO was used as a control. The percentage of drug remaining was determined by HPLC. The remaining concentration of drug was expressed as %drug remaining relative to the concentration of drug at zero time.

### 4.5. Cell Culture

Caco-2 cells (passage 35–45) were used in cellular studies. Cells were cultured in DMEM, supplemented with 10% FBS and 1% Penicillin/Streptomycin (PS) at 37 °C in an incubator maintained at 95% air, 5% CO_2_, and 95% humidity. Caco-2 cells were cultured in T75 flasks at a density of 1 × 10^6^ cells and were sub-cultured every 3 to 4 days, over cells reaching 80% to 90% confluency.

### 4.6. Cytotoxicity of AmB-Soluplus^®^ Micelles in Caco-2 Cells

Caco-2 cells were plated in a 96-well plate at a density of 2.5 × 10^4^ cells per well and allowed attachment overnight. Then, the cells were treated with AmB-PM diluted in HBSS to prepare samples of 5, 10, 15, 20, and 30 μg/mL, followed by incubation for 4 h at 37 °C in a CO_2_ incubator. Cells treated with Triton-X (0.1%) and HBSS were used as a negative control and positive control, respectively. After incubation, each medium containing sample was removed and washed with PBS followed by incubation for another 2 h with MTT stock solution (5 mg/mL in PBS). Media from all wells were removed, and DMSO added to dissolve the formazan crystals. The absorbance of dissolved formazan was measured at a wavelength of 540 nm by a microplate reader.

### 4.7. Cellular Uptake of AmB-Soluplus^®^ Micelles

Caco-2 cells at a density of 5 × 10^4^ cells were seeded in 24-well plates and allowed to attach for 24 h. The media were removed and washed with HBSS before testing. HBSS 1 mL containing AmB-PM (equivalent to 15 μg/mL of AmB) was added to each well and allowed to incubate for 30, 60, 120, 180, and 240 min. Free drug at the same concentration was used as a control. After the respective time points, the supernatant was discarded, and the cells were washed three times with ice-cold HBSS. Subsequently, 300 μL of ice-cold methanol was added and incubated at 4 °C for 1 h. Then, the extracted cells were collected and lysed using an ultrasonic bath followed by centrifugation at 12,000 rpm at 4 °C for 10 min. Finally, the supernatant was subjected to HPLC analysis for the quantification of AmB.

### 4.8. Cellular Transport of AmB-Soluplus^®^ Micelles Across Caco-2 Cell Monolayer

Caco-2 cells were seeded in a Transwell plate (Corning^®^, polyester membrane insert, 12 mm with a pore size of 0.4 μm) at densities of 1 × 10^5^ cells/well. The cells were cultivated for 14 to 21 days to allow them to reach the required confluent monolayers. The culture media were changed every 2 days until experiments were conducted. The integrity of the Caco-2 cell monolayers was confirmed by measuring the transepithelial electrical resistance (TEER) with an EVOM2 (World Precision Instruments, Sarasota, FL, USA) before use. However, only cell monolayers with TEER values more than 500 Ω.cm^2^ were used for transport studies. At the commencement of transport studies, the cellular monolayers were washed 3 times with warmed HBSS and allowed to equilibrate in the incubator for 30 min. Then, AmB-PM in HBSS, 0.4 mL, (equivalent to 15 μg/mL of AmB) was added to the apical chamber, while pre-warmed HBSS, 1.2 mL was added into each basolateral chamber. After incubation for 30, 60, 120, 180, and 240 min, 0.2 mL of basolateral medium was withdrawn and replaced with pre-warmed HBSS. Drug solutions in the collected samples were evaporated using the GeneVac EZ-2 evaporation system (GeneVac Ltd., Ipswich, UK) to increase drug concentrations. Collected samples were reconstituted with the mobile phase and centrifuged at 12,000 rpm for 10 min before HPLC analysis.

The transport of the drug from the apical to the basolateral chamber was represented as the amount of AmB transported versus time. In addition, the AmB apparent permeability coefficient (*P*_app_) was calculated according to the following equation:

(3) $$P_{\mathrm{app}}=\frac{\mathrm{dQ}/\mathrm{dt}}{C_{0}*A}\times 100$$

where dQ/dt is the slope of the cumulative drug permeated versus time curve (μg/h). A is the diffusion area (1.12 cm^2^), *C*_0_ is the initial concentration of AmB in the apical chamber.

At the end of each incubation time, samples were removed from the apical and basolateral chambers, and the cells were washed three times with warmed HBSS. All wells were filled with 0.5 and 1.5 mL standard growth media into apical and basolateral chambers, respectively, then incubated for 24 h. Finally, the integrity of the cell monolayer was determined by the TEER measurement.

### 4.9. Flow Cytometric Analysis (FACS) of Nile Red–Soluplus^®^ Micelles

Blank Soluplus^®^ micelles were labeled with the fluorescent probe, Nile red (40 μg stock solution in methanol) using a similar method as for AmB-PM. Briefly, Caco-2 cells at a density of 5 × 10^4^ cells were seeded in a 24-well plate and allowed to attach for 24 h. Following the removal of cell culture medium and washing with PBS, serum-free media, 1 mL, containing Nile red–Soluplus^®^ micelles (NR-PM) (equivalent to 2 μg/mL of Nile red) was added and incubated for 1, 2, and 4 h. At selected time intervals, the supernatants were discarded, and the remaining cells washed with ice-cold PBS three times. Cells were harvested with trypsin and washed using centrifugation at 1200 rpm for 5 min to obtain the cell pellets. Finally, cells were resuspended in cold FACS buffer and measured under flow cytometry (Becton Dickinson Pty Ltd., North Ryde, NSW, Australia). The mean fluorescent intensity of cells was analyzed with BD FACSDiva 8.0 software. Cells treated with culture media and Nile red were used as negative and positive controls, respectively.

### 4.10. Confocal Imaging of Nile Red–Soluplus^®^ Micelles

The uptake of NR-PM was visualized using confocal laser scanning microscopy (CLSM). Caco-2 cells at a density of 1 × 10^5^ cells were seeded on a coverslip over a 6-well plate and allowed to attach for 24 h. Cell culture media were removed, and cells were washed with PBS, followed by adding NR-PM (equivalent to 2 μg/mL of Nile red) and incubated for 1, 2, and 4 h. Afterward, cells were washed twice with PBS and 4% of paraformaldehyde was added in PBS to fix the cells and left to incubate overnight at 4 °C. The cells were washed twice with PBS and incubated with absolute ethanol for 20 min. Then, cells were washed three times and incubated with 700 μL of 1% bovine serum albumin solution in PBS for 15 min to block the non-specific binding sites. Alexa Fluor 488 conjugate was added and incubated for 30 min to stain the cytoplasm of cells, followed by washing twice with PBS. Finally, coverslips were collected and placed on glass slides containing 200 μL of anti-fade fluorescent mounting medium containing DAPI to stain the nucleus of cells. The cellular uptake of NR-PM was visualized under CLSM (Zeiss LSM 800, Carl-Zeiss, Oberkochen, Germany). ZEN Lite 2.6 software was used for image processing.

### 4.11. Statistical Analysis

All data are expressed as the mean ± standard deviation (SD). The statistical analysis was performed using one-way analysis of variance (ANOVA). Differences among treatment means within sampling periods were compared using Tukey’s post-hoc test. Differences with *p* < 0.05 were considered as statistically significant.

## 5. Conclusions

AmB-PM were successfully prepared by a modified solvent diffusion method using the NanoAssemblr^®^ Benchtop instrument, which uses laminar flow microfluidic mixing to achieve rapid and consistent particle quality, ease of use, reproducibility and is easy to scale up. The method allows rapid and cost-effective process optimization. AmB-PM were prepared based on amphiphilic properties of Soluplus^®^ to enhance the oral absorption of AmB. The drug was incorporated in the hydrophobic core and enclosed by a hydrophilic shell, which guarded the AmB against degradation in gastric fluid. The uptake and transport of AmB into Caco-2 cells was significantly increased by AmB-PM. AmB-PM (1:4) presented as the optimum formulation. It possessed a nanosize of ~80 nm and a high drug loading up to 20%, resulting in increased cellular uptake and enhanced transport efficiency crossing the intestinal epithelial cell monolayer through passive absorption and endocytosis. Considered overall, this study indicates the great potential of AmB-loaded Soluplus^®^ micelles for the oral delivery of AmB. There is certainly good justification for further work, including performance in animal studies and possible human studies.

## Supplementary Materials

The following are available online at https://www.mdpi.com/1424-8247/13/6/121/s1, Table S1: Mean particle size, charge, PDI, and %drug remaining of AmB-PM after 1 month at 4 °C in the absence of daylight. (Data are presented as mean ± SD; *n* = 3).
