# Supplementary material for: Copolymeric Micelles Overcome the Oral Delivery Challenges of Amphotericin B
Source: Pharmaceuticals (Basel). 2020 Jun 11;13(6):121. doi: 10.3390/ph13060121 (PMC7344903; doi:10.3390/ph13060121)
Supplement: Supplementary file 1 [file pharmaceuticals-13-00121-s001.pdf]

Table 1. Mean particle size, charge, PDI and %drug remaining of AmB-PM after 1 month at 4 °C in the absence of the day light. (Data were presented as mean ± SD; *n*=3).

	**AmB-PM (1:4)**		**AmB-PM (1:8)**	
	**Initial preparations**	**1 month at 4 °C**	**Initial preparations**	**1 month at 4 °C**
**Size (nm) ± SD**	85.4 ± 4.8	79.4 ± 3.5	84.0 ± 6.1	89.0 ± 2.1
**Charge (mV) ± SD**	7.27 ± 0.71	6.64 ± 0.22	6.77 ± 0.82	5.80 ± 0.44
**PDI ± SD**	0.10 ± 0.02	0.08 ± 0.02	0.072 ± 0.01	0.10 ± 0.01
**%Drug remaining ± SD**	100	93.78 ± 4.79	100	93.02 ± 6.09
